# 5-HT inhibition of rat insulin 2 promoter Cre recombinase transgene and proopiomelanocortin neuron excitability in the mouse arcuate nucleus

**DOI:** 10.1016/j.neuroscience.2008.12.003

**Published:** 2009-03-03

**Authors:** K. Hisadome, M.A. Smith, A.I. Choudhury, M. Claret, D.J. Withers, M.L.J. Ashford

**Affiliations:** a Biomedical Research Institute, Ninewells Hospital and Medical School, University of Dundee, Dundee, DD1 9SY, Scotland, UK; b Centre for Diabetes and Endocrinology, Rayne Institute, University College London, London, WC1E 6JJ UK

**Keywords:** 5-HT, hypothalamus, K^+^ channel, RIPCre, POMC, 5-HT_1F_ receptor, AgRP, agouti-related protein, ARC, arcuate nucleus, GFP, green fluorescent protein, LHA, lateral hypothalamic area, NPY, neuropeptide Y, PBG, 1-phenylbiguanide, POMC, proopiomelanocortin, PVN, paraventricular nucleus, RIPCre, rat insulin 2 promoter Cre recombinase transgene, TEA, tetraethylammonium chloride, TTX, tetrodotoxin, α-me 5-HT, α-methyl 5-HT, α-MSH, alpha-melanocyte stimulating hormone, ΔVm, change in membrane potential, 5-CT, 5-carboxamidotryptamine, 8-OH-DPAT, 8-hydroxy-2-(di-*n*-propylamino) tetralin

## Abstract

A number of anti-obesity agents have been developed that enhance hypothalamic 5-HT transmission. Various studies have demonstrated that arcuate neurons, which express proopiomelanocortin peptides (POMC neurons), and neuropeptide Y with agouti-related protein (NPY/AgRP) neurons, are components of the hypothalamic circuits responsible for energy homeostasis. An additional arcuate neuron population, rat insulin 2 promoter Cre recombinase transgene (RIPCre) neurons, has recently been implicated in hypothalamic melanocortin circuits involved in energy balance. It is currently unclear how 5-HT modifies neuron excitability in these local arcuate neuronal circuits. We show that 5-HT alters the excitability of the majority of mouse arcuate RIPCre neurons, by either hyperpolarization and inhibition or depolarization and excitation. RIPCre neurons sensitive to 5-HT, predominantly exhibit hyperpolarization and pharmacological studies indicate that inhibition of neuronal firing is likely to be through 5-HT_1F_ receptors increasing current through a voltage-dependent potassium conductance. Indeed, 5-HT_1F_ receptor immunoreactivity co-localizes with RIPCre green fluorescent protein expression. A minority population of POMC neurons also respond to 5-HT by hyperpolarization, and this appears to be mediated by the same receptor-channel mechanism. As neither POMC nor RIPCre neuronal populations display a common electrical response to 5-HT, this may indicate that sub-divisions of POMC and RIPCre neurons exist, perhaps serving different outputs.

The CNS control of food intake involves complex interactions between circulating hormones, nutrients, neuropeptides, monoamines and other neurotransmitters. These act at a variety of hypothalamic areas (including the paraventricular nucleus (PVN) and the lateral (LHA) and medial hypothalamic areas) to modulate orexigenic and anorexigenic neural pathways (Broberger, 2005). At least two populations of neurons within the arcuate nucleus (ARC) of the hypothalamus contribute to the central circuitry that controls energy homeostasis. These neurons make up part of the melanocortin pathway, which consists of cells containing neuropeptide Y (NPY) along with the endogenous melanocortin antagonist, agouti-related protein (AgRP) and cells containing alpha-melanocyte stimulating hormone (α-MSH) and other proopiomelanocortin (POMC) derived peptides (Ellacott and Cone, 2004; Cone, 2005). These neurons are key targets for the hormones leptin and insulin, the actions of which effect an anorexigenic output (Niswender et al., 2004).

Food intake is accompanied by changes in the release of monoamines in the hypothalamus (Schwartz et al., 1990), and sympathomimetic drugs (e.g. *d*-fenfluramine) have long been used to reduce food intake and appetite (Ioannides-Demos et al., 2005). Consequently, pharmacological manipulation that results in enhancement or inhibition of 5-HT synaptic transmission reduces and increases food intake, respectively, in animals and humans (Halford et al., 2005). Electrophysiological recordings from neurons of transgenic mice expressing green fluorescent protein (GFP) under the control of the POMC promoter demonstrate that 5-HT depolarizes arcuate POMC neurons (Heisler et al., 2002), an action also observed for leptin (Cowley et al., 2001; Choudhury et al., 2005). There are at least 14 different 5-HT receptor subtypes and many are present at significant levels in the hypothalamus, notably 5-HT_1B_, 5-HT_1F_, 5-HT_2A_, 5-HT_2B_, 5-HT_2C_ and 5-HT_7_ (Hoyer et al., 2002). However, it is still unclear exactly which 5-HT receptor subtypes contribute to modulation of activity in the hypothalamic circuits that sub-serve long-term control of food intake and energy expenditure. In addition, the underlying mechanisms by which 5-HT receptor activation alters the electrical activity of these ARC neurons are unknown. Studies, using selective 5-HT receptor subtype agonists and antagonists, have demonstrated both hyperphagic and hypophagic responses in animal studies. Unfortunately, many of these ligands lose their receptor selectivity at higher concentrations, resulting in some uncertainty over receptor subtype identity in relation to changes in food intake. Nevertheless, there are two main subtypes proposed as key mediators of the anorexigenic action of 5-HT, the 5-HT_1B_ and 5-HT_2C_ receptors (Ramos et al., 2005). Neurons expressing 5-HT_1B_ (Makarenko et al., 2002) and 5-HT_2C_ receptors (Clemett et al., 2000) are present in hypothalamic feeding centers (i.e. PVN, LHA and ARC), but are also found in brain areas not implicated in energy homeostasis (Hoyer et al., 2002). The 5-HT_2C_ receptor knockout mouse demonstrates increased food intake and obesity (Tecott et al., 1995) and the hypophagic action of *d*-fenfluramine is attenuated in the 5-HT_1B_ knockout mouse (Lucas et al., 1998). However, global 5-HT_1B_ and 5-HT_2C_ receptor knockout mice also develop physiological abnormalities such as seizures, anxiety and aggression (Tecott et al., 1995; Ramboz et al., 1996). These observations highlight the multi-functional role of the serotonergic system, but also raise the question whether the feeding behaviors observed are due to the lack of a given receptor in the energy regulatory centers and/or in areas associated with other physiological or pathological outcomes. In addition, although histochemical and *in situ* hybridization studies demonstrate that many 5-HT receptor subtypes are located in energy regulatory centers, these data provide no information about receptor-mediated alteration of neuronal function, most importantly in relation to changes in neuronal excitability.

Recent studies have indicated that another population of arcuate neurons, defined by GFP expression driven by the rat insulin 2 promoter Cre recombinase transgene (RIPCre), which are distinct from NPY/AgRP and POMC neurons, are involved in the regulation of body weight and energy homeostasis (Cui et al., 2004; Choudhury et al., 2005). Thus, we have examined the actions of 5-HT on the electrical activity of this population of arcuate neurons and show that they respond to 5-HT in a heterogeneous manner with the majority of responding neurons displaying hyperpolarization and reduced excitability.

## Experimental procedures

### Hypothalamic slice preparation

As previously described (Choudhury et al., 2005; Smith et al., 2007) we have used 2 Cre recombinase transgenic lines, RIPCre and POMCCre and inter-crossed these with the ZEG indicator mouse to generate mice with GFP expression in selective hypothalamic neuronal populations. All procedures conformed to the UK Animals (Scientific Procedures) Act 1986, and were approved by our institutional ethical review committee. Every effort was made to minimize the number of animals used and their suffering. RIPCreZEG and POMCCreZEG mice (8–16 weeks old) were killed by cervical dislocation; the brain was rapidly removed and submerged in an ice cold slicing solution containing (in mM) KCl 2.5, NaH_2_PO_4_ 1.25, NaHCO_3_ 28, CaCl_2_ 0.5, MgCl_2_ 7, d-glucose 7, ascorbate 1, pyruvate 3 and sucrose 235, equilibrated with 95% O_2_, 5% CO_2_ to give a pH of 7.4. Hypothalamic coronal slices (350 μm), containing the ARC, were cut using a Vibratome (St Louis, MO, USA), transferred and kept at room temperature (22–25 °C) in an external solution containing (in mM) NaCl 125, KCl 2.5, NaH_2_PO_4_ 1.25, NaHCO_3_ 25, CaCl_2_ 2, MgCl_2_ 1, d-glucose 10, d-mannitol 15, ascorbate 1 and pyruvate 3, equilibrated with 95% O_2_, 5% CO_2_, pH 7.4.

### Electrophysiology

Individual arcuate neurons were identified by epifluorescence and differential interference contrast optics using an upright Zeiss Axioskop-2 FS plus microscope. Slices were continually perfused with a modified external solution (0.5 mM CaCl_2_ and 2.5 mM MgCl_2_, no ascorbate and pyruvate) at a flow rate of 5–10 ml/min and a bath temperature of 33 °C. For high potassium experiments, the normal external solution was replaced with a solution containing (in mM) NaCl 130, KCl 20, CaCl_2_ 0.5, MgCl_2_ 2.5, d-glucose 10, d-mannitol 15, Hepes 10, pH 7.4. Patch-clamp recordings were performed using borosilicate patch pipettes (4–8 MΩ) filled with an internal solution containing (in mM) K-gluconate 130, KCl 10, EGTA 0.5, Hepes 10, NaCl 1, CaCl_2_ 0.28, MgCl_2_ 3, Na_2_ATP 3, tris-GTP 0.3, phosphocreatine 14 (pH 7.2). Whole-cell series resistance (Rs) was compensated using an Axopatch 200B amplifier (Molecular Devices, Sunnyvale, CA, USA) in current (I_fast_) and voltage-clamp modes (Rs: 30–60 and 10–30 MΩ respectively). Voltage and current commands were manually or externally driven using PClamp 9.2 software and injected into neurons via the patch-clamp amplifier. Under current clamp, hyperpolarizing current pulses (between −5 and −20 pA, at a frequency of 0.05 Hz) were used to monitor input and series resistance at resting membrane potentials. In addition, input resistance was calculated from I–V relationships evoked from a holding potential of −70 mV (±5–30 pA, 0.5 s pulse duration). Voltage clamp recordings of transient voltage-dependent potassium (*I*_A_) conductance and the delayed and inward rectifying potassium conductances were performed as described in Smith et al. (2007). Whole cell currents and voltages were filtered at 5 and 2 kHz respectively, and digitized at 50 kHz using Pclamp 9.2 software. All data were stored un-sampled on digital audiotape for off-line analysis using Clampex 9.2 or Igor pro. Membrane potentials were either replayed un-sampled on an EasyGraph TA240 chart recorder (Gould, Ballainvilliers, France), or digitized and imported into Abode illustrator for illustration purposes.

Drugs were added to the external solution and applied to slices via the perfusion system or locally applied using a broken tipped pipette (∼4 μm diameter) positioned above the recording neurons, as previously described (Choudhury et al., 2005). At least 10 min of stable control data were recorded before the application of any drug, and antagonists were applied for at least 10 min prior to challenge with agonist. Neuronal integrity was determined by biophysical and gross anatomical assessment, as described previously (Smith et al., 2007; Claret et al., 2007).

### Immunocytochemistry

Hypothalamic sections (30 μm) from paraformaldehyde perfused brains were processed as previously described (Choudhury et al., 2005). Primary antibodies used were rabbit polyclonal antibodies raised to the C- or N-terminal domains of the 5-HT_1F_ receptor and were obtained from MBL International (MA, USA; cat No. LS-3344 and LS-3338, respectively). Slices were incubated with primary antibody (1:300 dilution) for 48 h at 4 °C, following which they were incubated with anti-rabbit secondary antibody conjugated to Alexa Fluor 549 (1:800 dilution) for 1 h. RIPCre-GFP expression and 5-HT_1F_ receptor localization were detected using a confocal microscope (BioRad MRC 100).

### PCR

A 475 base pair fragment encoding a region of the 5-HT_1F_ receptor was detected by PCR from mRNA extracted from mouse hypothalamus using the following primers: forward GGAAGCTGAGTTGAGATGATGGC, reverse CACGTACAACAGATGATGTCG.

### Chemicals

Kynurenic acid, (+) bicuculline, tetraethylammonium chloride (TEA), 4-aminopyridine (4-AP), barium chloride, 5-HT, 8-hydroxy-2-(di-*n*-propylamino) tetralin (8-OH-DPAT), SB 242084 and CGS 12066B were purchased from Sigma-Aldrich (Dorset, UK). α-Methyl 5-HT (α-me 5-HT), 5-carboxamidotryptamine (5-CT), ketanserin, methiothepin, SB 204741, BW 723C86, CP 93129, SB 224289, L 694247 and BRL 54443 were obtained from Tocris Bioscience (Bristol, UK), tetrodotoxin (TTX) from Alomone Laboratories, Ltd. (Jerusalem, Israel), and zacopride from Professor B. Costell (University of Bradford). All drugs were dissolved in saline immediately before use.

### Statistical analysis

Responsive neurons were distinguished from non-responding neurons based on the criterion that the change in membrane potential (ΔVm) induced by the drug challenge was ±3 times the standard deviation of the mean membrane potential prior to addition of the drug (Smith et al., 2007; Claret et al., 2007). Consequently, a neuron was considered hyperpolarized or depolarized if the membrane potential was altered by ≥3 mV. Results are expressed as the mean±S.E.M. of the defined responses, with the number of cells studied. Statistical significance was determined on all neurons examined within a data set using a Student's two-tailed paired *t*-test or ANOVA, followed by Bonferroni's post hoc test, where appropriate. Comparisons between 5-HT ± agonist or 5-HT ± antagonist on neuron responses were made by paired two-tailed Student's *t*-test. A *P* value of less than 0.05 was considered statistically significant.

## Results

### 5-HT modulates the excitability of RIPCre neurons

**Fig. 1:**
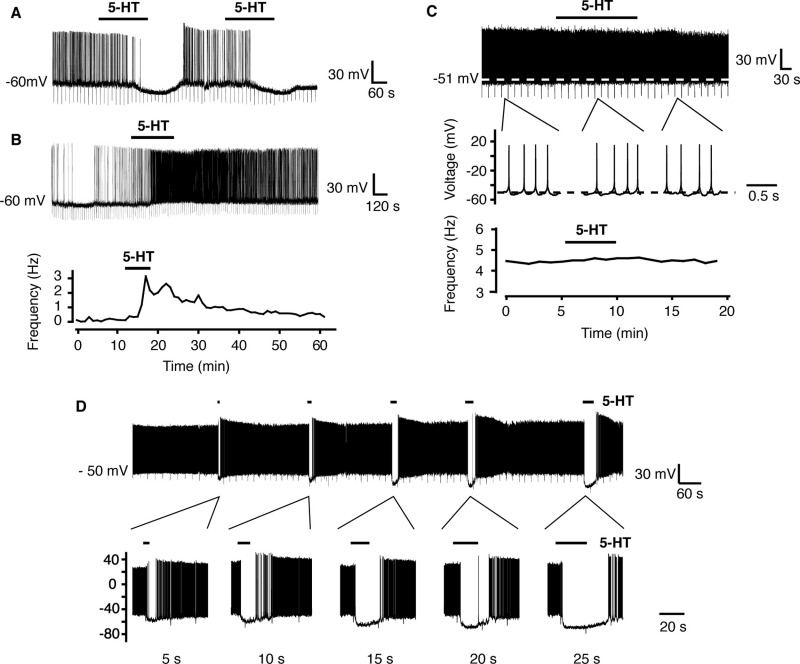
5-HT alters the excitability of RIPCre neurons. Whole-cell current clamp recordings were made from RIPCre neurons in the absence and presence of 5-HT. (A) The predominant response to bath applied 5-HT (1 μM) was hyperpolarization and inhibition of firing. This action of 5-HT was readily reversible and a second application of 5-HT produced the same effect. (B) A smaller proportion of RIPCre neurons responded to 5-HT by depolarization and increased excitability. A diary plot of firing frequency for this neuron is shown, with bath-applied 5-HT demonstrating a clear excitation. (C) The remaining proportion of RIPCre neurons tested was insensitive to 5-HT, with no evidence of a ΔVm or of firing frequency. Expanded regions of this recording are displayed to show more clearly that bath-applied 5-HT had no effect on membrane potential. The diary plot of firing frequency is shown for this neuron. (D) Increasing doses of 5-HT were locally-applied to a RIPCre neuron, shown previously to respond to 5-HT by hyperpolarization. Increasing the duration of pressure ejection of 5-HT (5 s–25 s) increased the magnitude and duration of the 5-HT response. Note that increasing the dose of 5-HT did not induce receptor desensitization at the time intervals used for successive 5-HT challenges, and there was no evidence for heterogeneity of response to 5-HT.

Whole-cell current-clamp recordings were made from RIPCre hypothalamic arcuate neurons identified by the expression of GFP. Under control recording conditions, and consistent with previous reports (Choudhury et al., 2005; Smith et al., 2007) RIPCre neurons were characterized (*n*=111) by a high mean input resistance, 1.5±0.1 GΩ, and spontaneously fired sodium mediated action potentials from a mean resting membrane potential of −57.0±0.5 mV at a frequency of 4.0±0.2 Hz. Changes in the electrical excitability of RIPCre neurons (as assessed by changes in membrane potential and firing frequency) were elicited by bath (1 μM) or locally (2–5 μM) applied 5-HT with no discernable difference in the output in relation to the method of application. Application of 5-HT resulted in clear heterogeneous responses in this population of neurons. Overall, 5-HT inhibited 50% (55/111), excited 25% (28/111) or did not alter the excitability of 25% (28/111) of RIPCre neurons. In neurons, which displayed an inhibitory response to 5-HT there was a rapid hyperpolarization (Fig. 1A) from a mean membrane potential of −50.6±0.8 mV to −63.3±1.1 mV (*n*=55, *P*<0.05), which resulted in a decrease in neuronal firing rate, from a mean value of 4.1±0.3 Hz to 0.6±0.2 Hz (*P*<0.05). This hyperpolarization and reduction in excitability was accompanied by a decrease in whole-cell input resistance from a mean value of 1.50±0.09 GΩ to 1.24±0.23 GΩ (*P*<0.05), indicating that an increase in conductance underlies this response. 5-HT-mediated hyperpolarizing or depolarizing neuronal responses (*n*=8) were also obtained (data not shown) from slices exposed to 0.5 μM TTX. RIPCre neurons that were excited by 5-HT, were rapidly depolarized (Fig. 1B) from a mean membrane potential of −52.4±1.1 mV to −47.2±1.2 mV (*n*=28, *P*<0.05), and this was accompanied by an increase in firing rate from a mean value of 3.1±0.5 Hz to 5.2±0.8 Hz (*P*<0.05). The depolarization and increased firing were not accompanied by any significant change in whole-cell input resistance (from 1.60±0.11 GΩ to 1.68±0.13 GΩ). In RIPCre neurons where electrical activity was unaffected by application of 5-HT (Fig. 1C), there was no significant change in either resting membrane potential (−50.0±0.9 mV to −50.4±0.9 mV) or firing rate (4.6±0.5 Hz to 4.9±0.6 Hz).

We found no evidence for any dose-dependent effect of 5-HT with respect to these alterations in excitability; RIPCre neurons responded to 5-HT by hyperpolarization, depolarization or no response regardless of concentration (up to 10 μM tested) or duration of 5-HT application. An example of such a single reproducible outcome is shown in Fig. 1D, where increasing amounts of 5-HT were pressure ejected (by altering the duration of pressure application) onto a RIPCre neuron, previously shown to respond to 5-HT by hyperpolarization. A dose-dependent increase in the hyperpolarizing response amplitude and duration was observed (*n*=3), with no evidence for the direction of the response being dependent upon the duration (dose) of 5-HT ejection. The changes in membrane potential and spike firing induced by 5-HT (and other agonists, see below) at the concentrations utilized in this study on RIPCre neurons were reversible on washout within 15–30 min. Moreover, no desensitization of response to 5-HT or agonists was observed when re-applied following several minutes of washout (e.g. Fig. 1A, D). In subsequent experiments, detailed below, selective agonists and antagonists were applied to RIPCre neurons following an initial 5-HT challenge, which was used to ascertain the category of the response (depolarizing, hyperpolarizing or non-responsive).

### 5-HT modulation of RIPCre neuron excitability is independent of 5-HT_2_ receptors

**Fig. 2:**
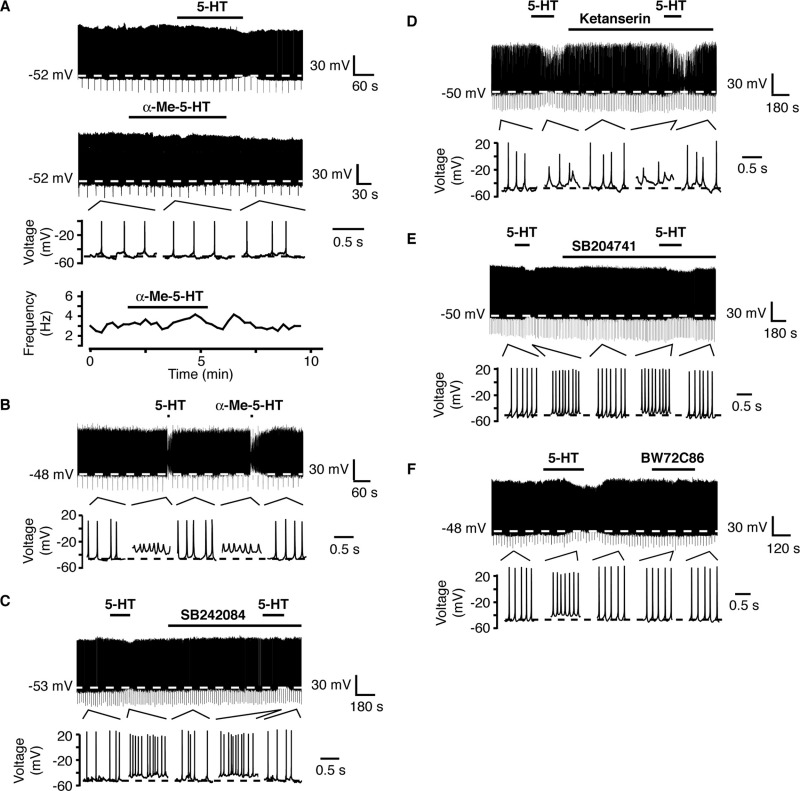
5-HT_2_ receptors are not responsible for 5-HT-induced RIPCre neuron depolarization. (A) Bath-applied α-me 5-HT (1 μM) did not affect the membrane potential or firing frequency of a RIPCre neuron, which had been shown previously to respond to 5-HT with depolarization (upper trace). Expanded traces and the corresponding diary plot of firing frequency for this neuron are shown. (B) Increasing the concentration of α-me 5-HT to 10 μM mimicked the depolarizing effect of 5-HT on the same RIPCre neuron. The expanded traces (lower) show the depolarization more clearly. Note that the depolarization of this neuron by either agonist was sufficient to cause severe action potential truncation. The presence of (C) the selective 5-HT_2C_ antagonist, SB242084 (100 nM) (D) ketanserin (10 nM) or (E) the 5-HT_2B_ antagonist SB204741 (100 nM) did not prevent locally-applied 5-HT from depolarizing and increasing the firing frequency of RIPCre neurons. (F) Application of 5 μM BW72C86, a selective 5-HT_2B_ agonist, had no effect on the excitability of RIPCre neurons that responded to 5-HT by depolarization.

A previous study has reported that 5-HT depolarizes POMC neurons via the activation of 5-HT_2C_ receptors (Heisler et al., 2002). To determine whether the depolarizing response observed in RIPCre neurons is also mediated by this receptor subtype, we first attempted to mimic the 5-HT depolarizing response on RIPCre neurons with a selective 5-HT_2_ receptor agonist, α-me 5-HT. At a concentration (1 μM) reported (Acuna-Castillo et al., 2002) to be relatively selective for 5-HT_2A/2C_ receptors, α-me 5-HT did not alter the excitability (Fig. 2A) of RIPCre neurons, shown to respond to 5-HT by depolarization (5-HT, ΔVm=+4.7±1.5 mV; α-me 5-HT, ΔVm=−1.5±0.8 mV, *n*=3, *P*<0.05) neurons. Increasing the concentration of α-me 5-HT to 10 μM resulted in depolarization (5-HT, ΔVm=+5.4±0.7 mV; α-me 5-HT, ΔVm=+6.6±2.2 mV; *n*=3) of RIPCre neurons (Fig. 2B), perhaps indicative of loss of selectivity to this agonist at a higher concentration. Therefore, we attempted to block the effects of 5-HT with selective receptor antagonists at concentrations where they have been reported to retain their selectivity. However, the presence of 100 nM SB242084, a selective 5-HT_2C_ receptor antagonist (Kennett et al., 1997b), failed (Fig. 2C) to prevent 5-HT from depolarizing RIPCre neurons (5-HT, ΔVm=+4.2±0.8 mV; 5-HT+SB242984, ΔVm=+6.5±1.7 mV, *n*=3). Furthermore, addition of 100 nM ketanserin to the bath solution, a concentration that will antagonize both 5-HT_2A_ and 5-HT_2C_ receptors (Baxter et al., 1995) also did not prevent 5-HT from depolarizing (Fig. 2D) RIPCre neurons (5-HT, ΔVm=+5.9±1.1 mV; 5-HT+ketanserin, ΔVm=+7.3±0.5 mV, *n*=3). The selective 5-HT_2B_ antagonist (Baxter et al., 1995), SB204741 (100 nM) was also incapable (Fig. 2E) of blocking the 5-HT depolarizing response in RIPCre neurons (5-HT, ΔVm=+5.8±1.6 mV; 5-HT+SB204741, ΔVm=+8.5±4.8 mV, *n*=3). The selective 5-HT_2B_ receptor agonist (Kennett et al., 1997a), BW723C86 (5 μM) was also unable to mimic (Fig. 2F) the 5-HT depolarization of RIPCre neurons (5-HT, ΔVm=+5.2±1.9 mV; BW723C86, ΔVm=−0.9±0.5 mV, *n*=3). The 5-HT_3_ receptor with its intrinsic non-selective cation channel, which depolarizes neurons when activated (Peters et al., 1992) is an attractive candidate to explain the 5-HT depolarization observed. However, bath application of the selective (Kilpatrick et al., 1990) 5-HT_3_ receptor agonist 1-phenylbiguanide (PBG; 1 μM), did not affect the resting membrane potential or firing frequency of RIPCre neurons (data not shown), previously demonstrated to respond to bath application of 5-HT by depolarization (5-HT, ΔVm=+5.7±1.2 mV; PBG, ΔVm=−0.54±1.3 mV, *n*=4).

**Fig. 3:**
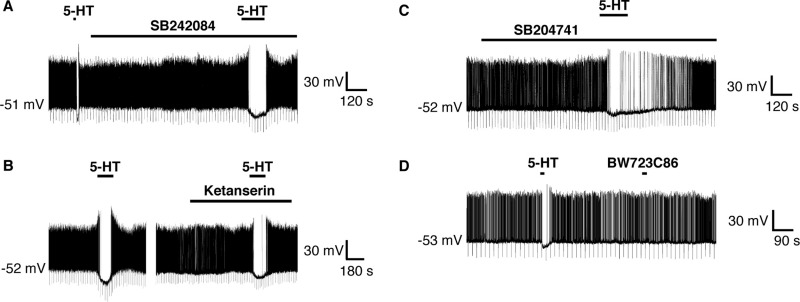
5-HT_2_ receptors do not mediate RIPCre neuron hyperpolarization by 5-HT (local application). The presence of (A) SB242084 (100 nM), (B) ketanserin (100 nM) or (C) SB204741 (100 nM) did not prevent 5-HT from hyperpolarizing RIPCre neurons. (D) Local application of BW723C86 (5 μM) did not mimic the hyperpolarizing action of 5-HT. The time gap in the recording shown in (B) is 38 min.

Similarly the hyperpolarizing response elicited by 5-HT in RIPCre neurons was not mediated by a 5-HT_2_ receptor subtype. Thus, in RIPCre neurons inhibited by 5-HT, the presence of 100 nM SB242084 (5-HT, ΔVm=−16.9±1.4 mV; 5-HT+SB242084, ΔVm=−16.6±2.1 mV, *n*=3), 100 nM ketanserin (5-HT, ΔVm=−15.0±2.9 mV; 5-HT+ketanserin, ΔVm=−14.5±3.4 mV, *n*=3) or 100 nM SB204741 (5-HT, ΔVm=−17.1±5.2 mV; 5-HT+SB204741, ΔVm=−20.4±4.4 mV, *n*=3) did not reduce the hyperpolarizing response induced by local application of 5-HT (Fig. 3A–C). Additionally, the 5-HT_2B_ selective agonist, BW723C86 (5 μM) was also unable to mimic the 5-HT hyperpolarization response (Fig. 3D) on RIPCre neurons (5-HT, ΔVm=−14.1±6.8 mV; BW723C86, ΔVm=−2.0±1.8 mV, *n*=3). Overall, these data indicate that 5-HT utilizes 5-HT receptors other than those of the 5-HT_2_ receptor subtype to elicit modification of RIPCre neuronal excitability.

### 5-HT_1_ receptors mediate hyperpolarization of RIPCre neurons

**Fig. 4:**
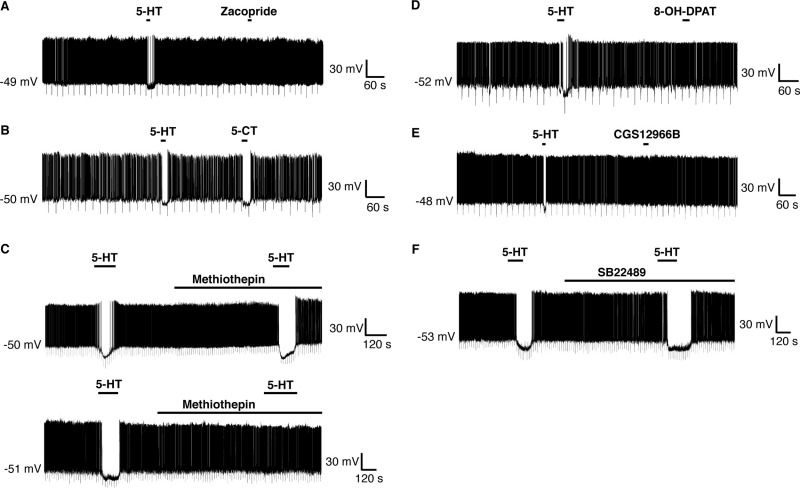
5-HT_1_ receptors mediate RIPCre neuron hyperpolarization by 5-HT. (A) Zacopride (2 μM) could not mimic the hyperpolarization of RIPCre neurons by 5-HT. (B) Local application of 5-CT (1 μM) hyperpolarized and inhibited the firing of RIPCre neurons, in a manner identical to that of 5-HT. (C) 5-HT-mediated hyperpolarization of RIPCre neurons was insensitive to block by 200 nM methiothepin (upper trace), but could be prevented by 2 μM methiothepin (lower trace). Local application of (D) 8-OH DPAT (10 μM) or (E) CGS12066B (1 μM) was unable to mimic the hyperpolarizing action of 5-HT. (F) The presence of SB22489 (100 nM) did not prevent 5-HT from hyperpolarizing RIPCre neurons.

As the hyperpolarizing response to 5-HT on RIPCre neurons was the dominant outcome electrically, we decided to focus on this response. Consequently we examined, using selective pharmacology, which of the remaining 5-HT receptor subtypes was likely responsible. Zacopride is a 5-HT_3_ receptor antagonist and a 5-HT_4_ receptor agonist (Schiavi et al., 1994). However, RIPCre neurons (Fig. 4A) that were hyperpolarized by 5-HT, did not respond to 100 μM zacopride (5-HT, ΔVm=−7.9±3.1 mV; zacopride, ΔVm=−1.8±2.1 mV, *n*=3). In contrast, application of the mixed 5-HT_1,5,7_ receptor agonist 5-CT (2 μM) reversibly hyperpolarized (Fig. 4B) RIPCre neurons (5-HT, ΔVm=−11.6±0.6 mV; 5-CT, ΔVm=−13.3±2.2 mV, *n*=4). In an attempt to distinguish between the 5-HT_1,5,7_ receptor subtypes, we examined the responsiveness of RIPCre neurons to 5-HT in the presence of 200 nM methiothepin (a concentration expected to bind and antagonize 5-HT_2A,2C_ and 5-HT_5,6,7_ receptor subtypes; Hoyer et al., 1994). However, this concentration of methiothepin had no effect (Fig. 4C) on the inhibitory action of 5-HT on RIPCre neurons (5-HT, ΔVm=−14.7±5.9 mV; 5-HT+methiothepin, ΔVm=−13.9±5.6 mV, *n*=4). In contrast, 2 μM methiothepin, (Fig. 4C) completely inhibited the hyperpolarizing action of 5-HT on these neurons (5-HT, ΔVm=−11.9±3.4 mV; 5-HT+methiothepin, ΔVm=−0.7±0.5 mV, *n*=4). Consequently, these data point toward the 5-HT_1_ receptor subtype family as likely responsible for the 5-HT-mediated hyperpolarization of RIPCre neurons.

**Fig. 5:**
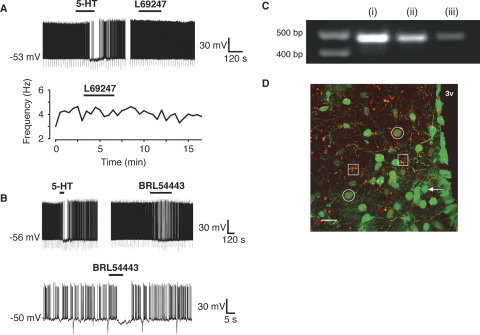
RIPCre neurons are hyperpolarized by BRL54443, a 5-HT_1E/F_ agonist and express 5-HT_1F_ receptors. (A) The 5-HT_1D_ receptor agonist, L69247 (1 nM) did not alter RIPCre neuron membrane potential or firing frequency. The gap in the recording shown is 17 min. (B) BRL54443 (20 nM) hyperpolarized and inhibited the firing of RIPCre neurons that responded by hyperpolarization to 5-HT. The gap in the recording (upper trace) is 22 min. (C) RT-PCR detection of the 5-HT_1F_ receptor transcript in (i) whole mouse brain and (ii), (iii) in separate mouse hypothalami. (D) Stacked (30 μm) confocal image of the ARC captured in 1 μm serial sections. Dual fluorescence for GFP (green) and 5-HT_1F_ receptor (N-terminal domain antibody: red), 5-HT_1F_ receptors can be observed on both GFP-positive (white circles) and GFP-negative (white squares) cells, although a minority of GFP-positive neurons display little or no immunoreactivity (e.g. white arrow). Note the C-terminal domain 5-HT_1F_ receptor antibody produced similar images (data not shown). 3v, Third ventricle. Scale bar=20 μm in (D). For interpretation of the references to color in this figure legend, the reader is referred to the Web version of this article.

It has previously been demonstrated that 5-HT_1A_ receptor activation induces hyperpolarization of hypothalamic (Muraki et al., 2004) and dorsal raphe (Katayama et al., 1997) neurons. However, application of the 5-HT_1A_ receptor-preferential agonist, 8-OH-DPAT, which also acts as a 5-HT_5_ receptor agonist at the concentrations employed here (10 μM), did not mimic (Fig. 4D) 5-HT-induced hyperpolarization of RIPCre neurons (5-HT, ΔVm=−8.4±3.4 mV; 8-OH-DPAT, ΔVm=−1.4±0.7 mV, *n*=3). 5-HT_1B_ receptor knockout mice have been reported to display reduced sensitivity to the anorexigenic actions of *d*-fenfluramine (Lucas et al., 1998). The selective rodent 5-HT_1B_ receptor agonist CGS12066B (1 μM) did not induce RIPCre neuron hyperpolarization (5-HT, ΔVm=−9.2±3.3 mV; CGS12066B, ΔVm=−0.8±0.5 mV, *n*=3) (Fig. 4E). Moreover, the presence of SB224289, at a concentration (100 nM) that specifically antagonizes 5-HT_1B_ receptors (Selkirk et al., 1998) was unable to prevent 5-HT induced hyperpolarization (5-HT, ΔVm=−17.7±3.5 mV; 5-HT+SB224289, ΔVm=−16.7±2.6 mV, *n*=3; Fig. 4F) of RIPCre neurons. Consequently, we examined other 5-HT_1_ receptor subtype-specific agonists. The selective 5-HT_1D_ agonist (Beer et al., 1993), L694247 (2 nM), had no effect on RIPCre neuron membrane potential (5-HT, ΔVm=−10.3±2.5 mV; L694247, ΔVm=+1.1±0.5 mV, *n*=3; Fig. 5A). In contrast, the mixed 5-HT_1E,1F_ receptor agonist BRL54443 at a concentration (20 nM) that retains selectivity for these subtypes (Brown et al., 1998) hyperpolarized (Fig. 5B) RIPCre neurons (5-HT, ΔVm=−11.0±2.1 mV, BRL54443, ΔVm=−5.3±0.8 mV, *n*=5). There is no evidence for 5-HT_1E_ receptor expression in mouse (Bai et al., 2004) and our PCR analysis demonstrates the presence of 5-HT_1F_ mRNA in mouse hypothalamic tissue blocks (Fig. 5C). Thus, in order to corroborate the presence of 5-HT_1F_ receptors in mouse arcuate neurons, we performed immunohistochemistry for 5-HT_1F_ receptors. In control experiments, both 5-HT_1F_ receptor antibodies displayed immunoreactivity in various brain regions, but not in heart, consistent with the expression profile for this receptor (Lovenberg et al., 1993). Using hypothalamic slices from RIPCreGFP mice and two different 5-HT_1F_ receptor antibodies (raised to separate epitopes of this receptor), 5-HT_1F_ receptors were demonstrated to be present in the hypothalamus, with high levels in the ARC (Fig. 5D). As is expected for membrane receptors, 5-HT_1F_ immunoreactivity appeared as small (<1 μm) distinct puncta that co-localized with the plasma membrane on the soma and neurites. Many RIPCreGFP positive neurons expressed 5-HT_1F_ receptors (circled in Fig. 5D), although occasional GFP positive neurons without significant 5-HT_1F_ staining could also be observed (e.g. white arrow in Fig. 5D), consistent with the notion that a significant sub-population of RIPCre neurons does not respond to 5-HT by hyperpolarization. In addition, numerous non-GFP cells in the ARC showed significant staining for the 5-HT_1F_ receptor (e.g. squares in Fig. 5D), supporting the view that 5-HT_1F_ receptor expression is not limited to a single neuronal phenotype.

### BRL54443 inhibits RIPCre neurons by increasing a voltage-dependent K^+^ conductance

**Fig. 6:**
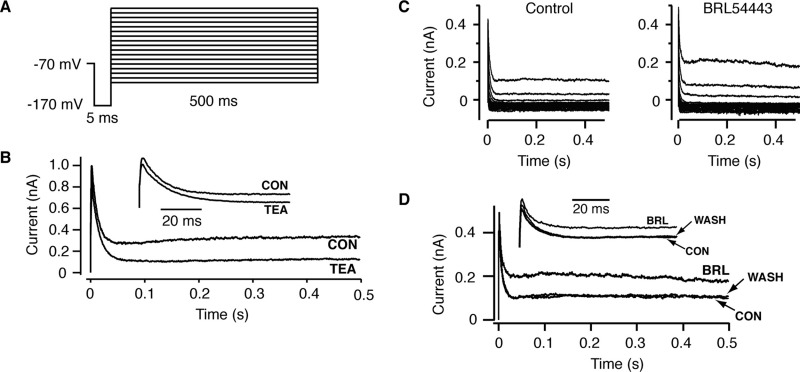
BRL54443 increases I_K_, a delayed rectifier-type K^+^ current, in RIPCre neurons. (A) Voltage clamp pulse protocol for recording of potassium currents. The 5 ms pre-pulse to −170 mV was used to deactivate any residual voltage dependent potassium conductance. (B) Representative currents from a RIPCre neuron elicited by a −10 mV voltage step in the absence and presence of bath-applied TEA (40 mM). Inset traces show the relatively small effect of TEA on the transient, peak potassium current. (C) Representative current families in the absence and presence of BRL54443 (20 nM). (D) Expanded single test pulses to −10 mV, showing that BRL54443 increased the steady-state current amplitude, which was reversed on washout, but had only a small effect on the peak transient current amplitude (inset expanded traces).

In an attempt to identify the conductance(s) modulated by BRL54443 that give rise to the hyperpolarizing response, RIPCre neurons were voltage-clamped in an external solution containing 10 μM bicuculline, 2 mM kynurenic acid and 1 μM TTX to block synaptic transmission and regenerative Na^+^ spikes. Neurons were held at −70 mV and voltage pulses (500 ms duration) were stepped from −90 to −10 mV in 5 mV increments, with a 5 ms pre-pulse stepped to −170 mV to deactivate voltage dependent potassium conductances (Fig. 6A). As described for POMC (Roseberry et al., 2004) and RIPCre (Smith et al., 2007) neurons, the inward rectifier potassium (K_IR_) conductance in arcuate neurons is extremely small under our control recording conditions. To increase the magnitude and induce a shift in the reversal potential (to approximately −50 mV) of the K_IR_ conductance, the external potassium concentration was raised from 2.5–20 mM (Smith et al., 2007). We have previously shown (Smith et al., 2007), for RIPCre neurons, that the K_IR_ conductance is blocked by 100 μM Ba^2+^, the transient voltage-dependent potassium (I_A_) conductance by 4 mM 4-aminopyridine, and the delayed rectifier type voltage-dependent potassium conductance partly blocked by 40 mM external TEA (see also Fig. 6B). Use of these blockers allowed identification and relative isolation of the main potassium currents observed in these neurons under our recording conditions. Local application of 20 nM BRL54443 to RIPCre neurons had no effect on K_IR_, with a linear slope conductance (measured between −90 and −50 mV) in 20 mM [K_o_] of 1.6±0.5 nS in control and 1.6±0.5 nS (*n*=8) in the presence of BRL54443 (data not shown). Similarly, 20 nM BRL54443 did not significantly change the peak amplitude of I_A_ (measured at −10 mV; 112±6% of control, *P*>0.05, *n*=8). In contrast, 20 nM BRL54443 reversibly increased the steady state depolarization-activated potassium current (measured at the end of the −10 mV command pulse) by 36±8% (*P*<0.05, *n*=8, Fig. 6C, D).

### Hyperpolarization of POMC neurons

**Fig. 7:**
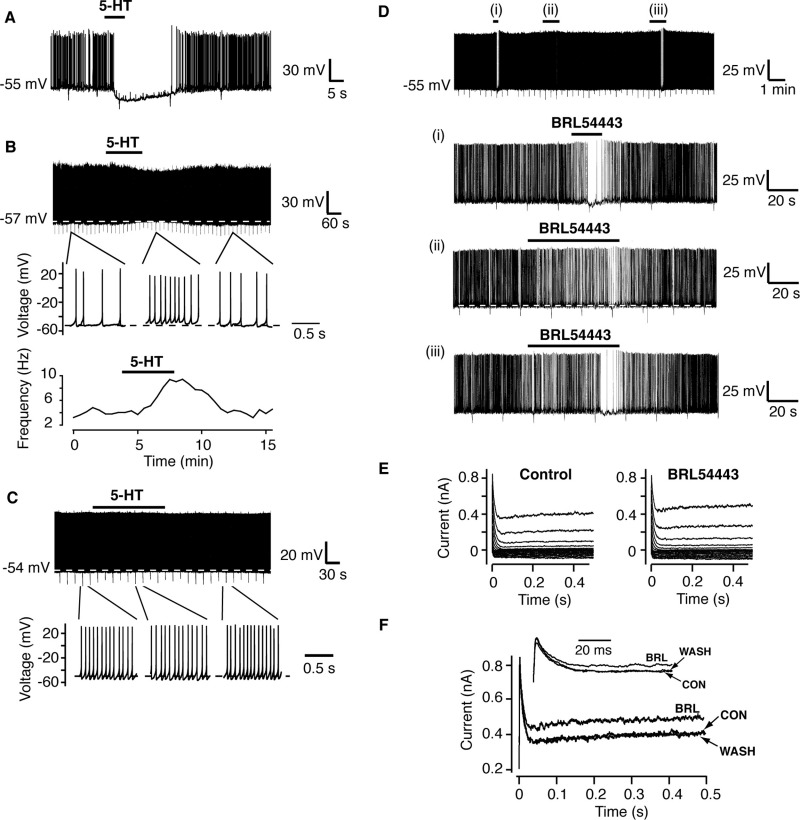
POMC neurons exhibit heterogeneous electrical responses to 5-HT. Example of a POMC neuron hyperpolarized and inhibited (A) or depolarized and excited (B) by locally-applied 5-HT. Expanded traces and the corresponding diary plot of firing frequency for this neuron are shown. (C) The majority of POMC neurons, challenged with 5-HT (local application), exhibited no ΔVm or of firing frequency, as shown in the expanded traces. (D) Example of BRL54443 (20 nM) hyperpolarization of a POMC neuron. Expanded traces are shown below for each agonist application ((i)–(iii)). Note that a second application of BRL54443 (20 nM) ∼3 min after the first was unable to elicit a response, and even after ∼9 min an increased dose of BRL54443 was required to produce significant hyperpolarization and reduction in firing. (E, F) BRL54443 reversibly increased the amplitude of the steady-state potassium current in POMC neurons. Representative current families in the absence and presence of BRL54443 (20 nM) are shown (E) with expanded single test pulses to −10 mV (F) to demonstrate this action of BRL54443 is reversible and limited to the steady-state, not the peak, potassium current amplitude.

Whole-cell current clamp recordings were made from POMC neurons, identified by expression of GFP, under the same conditions used for RIPCre neuron recordings. POMC neurons had a high mean input resistance of 1.4±0.1 GΩ and spontaneously fired action potentials from a mean resting membrane potential of −53.9±0.9 mV (*n*=40). As observed for RIPCre neurons, local application of 5-HT (2 μM) resulted in heterogeneous responses from POMC neurons, with 25% depolarized (10/40), 12.5% hyperpolarized (5/40) and the remainder 62.5% (25/40) unaffected by this concentration of 5-HT. In neurons, which displayed an inhibitory response to 5-HT there was a rapid hyperpolarization (Fig. 7A) from a mean membrane potential of −53.2±2.9 mV to −61.5±5.4 mV (*n*=5, *P*<0.05), which resulted in a decrease in neuronal firing rate, from a mean value of 3.3±0.5 Hz to 0.5±0.3 Hz (*P*<0.05). POMC neurons excited by 5-HT, were rapidly depolarized (Fig. 7B) from a mean membrane potential of −55.9±2.5 mV to −48.3±4.0 mV (*n*=10, *P*<0.05). In POMC neurons unaffected by application of 5-HT (Fig. 7C), there was no significant change in either resting membrane potential (−52.6±0.9 mV to −52.7±0.9 mV) or firing rate (3.9±0.5 Hz to 4.4±0.7 Hz).

In order to determine whether the 5-HT-mediated hyperpolarization of POMC neurons was mediated by the same receptor-channel mechanism as described above for RIPCre neurons, we examined the actions of BRL54443 on POMC neuron excitability. Local application of BRL54443 (20 nM) transiently hyperpolarized (ΔVm=−5.5±1.5 mV, *P*<0.05) and reduced the firing rate (Control=12.5±5.0 Hz; BRL54443=5.9±3.6 Hz, *P*<0.05) of POMC neurons (*n*=4). Successive application of BRL54443 produced an identical hyperpolarizing response on the same POMC neuron (Fig. 7D), although, in contrast to RIPCre neurons, POMC neurons displayed desensitization to BRL54443 (Fig. 7D) and 5-HT (data not shown), on re-application of agonists within 5 min of the first agonist challenge. Under the same voltage clamp conditions described for RIPCre neurons, BRL54443 (20 nM) reversibly increased the steady-state depolarization-activated conductance (measured at −10 mV) in POMC neurons (Fig. 7E, F) by 33±10% (*P*<0.05, *n*=4), with no change in K_IR_ slope conductance (1.5±0.4 versus 1.6±0.5 nS, *n*=4) or I_A_ amplitude (102±8% of control, *P*>0.1, *n*=4). These data indicate that 5-HT_1F_ receptor activation, resulting in an increased voltage-dependent potassium conductance, may underlie the 5-HT-induced hyperpolarization of POMC neurons described above.

## Discussion

The primary aim of this study was to examine the effect of 5-HT on RIPCre neuron electrical excitability. It had previously been reported that 5-HT_2C_ receptor agonists could depolarize and excite POMC neurons (Heisler et al., 2002). Our results showed that although RIPCre neurons could be depolarized by 5-HT this was not the majority response, with only 25% demonstrating an increase in excitability. In fact, half of all neurons tested displayed a hyperpolarizing, inhibitory, response to 5-HT application. The 5-HT induced changes in excitability of RIPCre neurons are likely to be predominantly directly mediated rather than synaptically driven as similar responses are observed in the presence of TTX in current clamp recordings. The increased voltage-dependent potassium currents observed in voltage-clamped RIPCre and POMC neurons in the presence of TTX and GABA and glutamate receptor antagonists are also consistent with a direct effect of 5-HT on these neurons. Previous studies of POMC and RIPCre neurons have demonstrated that peptide agonists and hormones do not elicit responses from every identified neuron (Cowley et al., 2001; Choudhury et al., 2005; Plum et al., 2006; Smith et al., 2007; Claret et al., 2007; Könner et al., 2007). It was expected that 5-HT would alter the excitability of RIPCre neurons by one or more of the following receptor subtypes; 5-HT_1A,B_, 5-HT_2A,B,C_ or 5-HT_7_, as these have been shown to be expressed in the medial hypothalamus or arcuate, and implicated in 5-HT modulation of feeding (Hoyer et al., 2002; Ramos et al., 2005). Indeed, we reasoned that RIPCre neuron depolarization could occur through the same receptor mechanism reported for POMC neurons. However, surprisingly we were unable to demonstrate, using selective 5-HT_2A,2B,2C_ antagonists and a selective 5-HT_2B_ agonist that the 5-HT-induced depolarization of RIPCre neurons was due to any of these subtypes. Clearly the receptor subtype and identity of the channel mechanisms underlying the 5-HT depolarization of RIPCre neurons will require further analysis.

5-HT-induced hyperpolarization of RIPCre neurons was unlikely to be mediated by a member of the 5-HT_2_ family as α-me 5-HT was ineffective, the 5-HT responses were insensitive to 5-HT_2A,B,C_ antagonists and a selective 5-HT_2B_ agonist. In contrast, the observation that 5-CT hyperpolarized RIPCre neurons suggested that the receptor underlying the 5-HT hyperpolarization is a member of the 5HT_1_ family, or the 5-HT_5_ or 5-HT_7_ receptor. However, the lack of effect of 8-OH-DPAT, CGS12066B and L694247 as agonists and SB 224289 and sub-micromolar methiothepin as antagonists to 5-HT-mediated hyperpolarization, effectively ruled out 5-HT_1A,B,D_ and 5-HT_5,7_ receptors as responsible. The observation that low concentrations (10 or 20 nM) of the mixed 5-HT_1E,F_ receptor agonist, BRL54443 hyperpolarized and that micromolar concentrations of methiothepin prevented 5-HT induced hyperpolarization of RIPCre neurons indicates that one of these subtypes is likely responsible. Unfortunately, no selective 5-HT_1F_ receptor agonist or antagonist was available to allow us to distinguish between these 5-HT receptor isoforms, but as mice do not possess the gene for 5-HT_1E_ receptors (Bai et al., 2004) we suggest that the 5-HT-induced hyperpolarization of RIPCre neurons is mediated by activation of the 5-HT_1F_ receptor. The human cloned 5-HT_1F_ receptor has previously been demonstrated to require a methiothepin concentration of ∼1 μM to inhibit a functional response (Adham et al., 1993). Previous studies have indicated that mRNA for the 5-HT_1F_ receptor is present in human brain (Hoyer et al., 1994, 2002), and *in situ* hybridization studies of mouse brain indicate its presence in numerous regions, including the hypothalamus (Bonnin et al., 2006). In support of this contention, mRNA for the 5-HT_1F_ receptor was detected by PCR in mouse hypothalamus in agreement with a previous study in rat (Lovenberg et al., 1993). Furthermore, immunohistochemical analysis of mouse ARC slices, using two separate antibodies to the 5-HT_1F_ receptor demonstrated its expression in most RIPCre neurons as well as other, undefined, neurons in the ARC. BRL54443 increased current through a channel that has the basic characteristics of a steady-state, delayed rectifier-like potassium channel with no effect on K_IR_ or I_A_, the other major potassium currents present in these neuron types (Smith et al., 2007). Previous studies have demonstrated that a number of 5-HT receptor subtypes are capable of coupling to K^+^ channels although this appears primarily mediated via Ca^2+^-activated K^+^ channels or G-protein-gated inwardly rectifying K^+^ channels (Raymond et al., 2001), rather than the delayed rectifier family of K^+^ channels. Although we demonstrate that BRL54443 increases the current through a voltage-gated potassium conductance, it is unlikely that the typical delayed rectifier or I_A_ potassium channels (i.e. Kv1–4 families) underlie the 5-HT-mediated hyperpolarization. 5-HT and BRL54443 hyperpolarize RIPCre and POMC neurons from a membrane potential of −50 to −55 mV, a range where most delayed rectifier channels are closed. One plausible candidate group of voltage-gated potassium channels is the KCNQ gene family. These encode Kv7 channel subunits, which are expressed widely in the brain, exhibit voltage dependent activation, are functionally active at the resting membrane potential of many neurons and can be modulated by neurotransmitters (Hansen et al., 2008). Additionally, the coupling mechanism by which this 5-HT receptor increases potassium channel current is presently unknown. Studies of the human 5-HT_1F_ receptor, expressed in 3T3 or fibroblast cell lines, indicate that this receptor subtype is capable of engaging with multiple cell signal transduction pathways (i.e. cAMP, inositol phosphates and intracellular calcium), in a cell-specific manner (Adham et al., 1993).

Our results do support the contention that at least part of the anorexigenic actions of 5-HT in the hypothalamus may be via the central melanocortin system (Heisler et al., 2002, 2006). Indeed, the main action of 5-HT on identified POMC neurons, albeit on a minority of the population (25%) we sampled in the arcuate, was depolarization and increased excitation in agreement with the previous report (Heisler et al., 2002). This result is consistent with the report that low concentrations of 5-HT directly stimulate α-MSH release from POMC neurons in the hypothalamus (Tiligada and Wilson, 1989). However, POMC neurons can also respond to 5-HT by hyperpolarization and inhibition of firing, and our current clamp and voltage clamp data using BRL54443 as an agonist, suggest this outcome may also be driven by the same 5-HT receptor-voltage-activated potassium channel mechanism described for ARC RIPCre neurons. Thus, POMC neurons also appear capable of divergent electrical responses to 5-HT and this may be linked to differential outputs, such as altered food intake and energy expenditure or modulation of peripheral glucose levels or fat storage, sub-served by different groups of POMC neurons (Cone, 2005). Indeed, there is additional evidence that multiple 5-HT-dependent mechanisms contribute to modulation of POMC neuron excitability as Heisler et al. (2006) have shown that 5-HT, by activating 5-HT_1B_ receptors on NPY/AgRP neurons, can reduce the inhibitory drive onto POMC neurons (i.e. indirect excitation).

The responses of the RIPCre neurons investigated here indicate that the predominant action of 5-HT is to hyperpolarize, and inhibit, these neurons. This occurs through activation of a voltage-gated K^+^ conductance and is likely mediated by the 5-HT_1F_ receptor subtype. Unfortunately, it is not yet possible to judge the physiological significance of this inhibition of RIPCre neuron function, or its potential relation to 5-HT-mediated physiological outcomes such as reduced food consumption or mood alteration. This is because we do not presently know the identity of the transmitter(s)/peptide(s) released from this neuronal population, nor how these neurons relate to the NPY/AgRP and POMC neuron ARC circuitry, although there is evidence to suggest that RIPCre neurons may be part of the melanocortin system (Choudhury et al., 2005; Smith et al., 2007).

## References

[bib1] Acuna-Castillo C., Villalobos C., Moya P.R., Saez P., Cassels B.K., Huidobro-Toro J.P. (2002). Differences in potency and efficacy of a series of phenylisopropylamine/phenylethylamine pairs at 5-HT(2A) and 5-HT(2C) receptors. Br J Pharmacol.

[bib2] Adham N., Borden L.A., Schechter L.E., Gustafson E.L., Cochran T.L., Vaysse P.J., Weinshank R.L., Branchek T.A. (1993). Cell-specific coupling of the cloned human 5-HT1F receptor to multiple signal transduction pathways. Arch Pharmacol.

[bib3] Bai F., Yin T., Johnstone E.M., Su C., Varga G., Little S.P., Nelson D.L. (2004). Molecular cloning and pharmacological characterization of the guinea pig 5-HT1E receptor. Eur J Pharmacol.

[bib4] Baxter G., Kennett G., Blaney F., Blackburn T. (1995). 5-HT2 receptor subtypes: a family re-united?. Trends Pharmacol Sci.

[bib5] Beer M.S., Stanton J.A., Bevan Y., Heald A., Reeve A.J., Street L.J., Matassa V.G., Hargreaves R.J., Middlemiss D.N. (1993). L-694,247: a potent 5-HT1D receptor agonist. Br J Pharmacol.

[bib6] Bonnin A., Peng W., Hewlett W., Levitt P. (2006). Expression mapping of 5-HT1 serotonin receptor subtypes during fetal and early postnatal mouse forebrain development. Neuroscience.

[bib7] Broberger C. (2005). Brain regulation of food intake and appetite: molecules and networks. J Intern Med.

[bib8] Brown A.M., Avenell K., Young T.J., Ho M., Porter R.A., Vimal M., Middlemiss D.N. (1998). BRL 54443, a potent agonist with selectivity for cloned 5-HT1E and 5-HT1F receptors. Br J Pharmacol.

[bib9] Choudhury A.I., Heffron H., Smith M.A., Al-Qassab H., Xu A.W., Selman C., Simmgen M., Clements M., Claret M., MacColl G., Bedford D.C., Hisadome K., Diakonov I., Moosajee V., Bell J.D., Speakman J.R., Batterham R.L., Barsh G.S., Ashford M.L.J., Withers D.J. (2005). The role of insulin receptor substrate 2 in hypothalamic and β cell function. J Clin Invest.

[bib10] Claret M., Smith M.A., Batterham R.L., Selman C., Choudhury A.I., Fryer L.G.D., Clements M., Al-Qassab H., Heffron H., Xu A.W., Speakman J.R., Barsh G.S., Viollet B., Vaulont S., Ashford M.L.J., Carling D., Withers D.J. (2007). AMPK is essential for energy homeostasis regulation and glucose-sensing by POMC and AgRP neurons. J Clin Invest.

[bib11] Clemett D.A., Punhani T., Duxon M.S., Blackburn T.P., Fone K.C. (2000). Immunohistochemical localization of the 5-HT2C receptor protein in the rat CNS. Neuropharmacology.

[bib12] Cone R.D. (2005). Anatomy and regulation of the central melanocortin system. Nat Neurosci.

[bib13] Cowley M.A., Smart J.L., Rubinstein M., Cerdán M.G., Diano S., Horvath T.L., Cone R.D., Low M.J. (2001). Leptin activates anorexigenic POMC neurons through a neural network in the arcuate nucleus. Nature.

[bib14] Cui Y., Huang L., Elefteriou F., Yang G., Shelton J.M., Giles J.E., Oz O.K., Pourbahrami T., Lu C.Y.H., Richardson J.A., Karsenty G., Li C. (2004). Essential role of STAT3 in body weight and glucose homeostasis. Mol Cell Biol.

[bib15] Ellacott K.L., Cone R.D. (2004). The central melanocortin system and the integration of short- and long-term regulators of energy homeostasis. Recent Prog Horm Res.

[bib16] Halford J.C., Harrold J.A., Lawton C.L., Blundell J.E. (2005). Serotonin (5-HT) drugs: effects on appetite expression and use for the treatment of obesity. Curr Drug Targets.

[bib17] Hansen H.H., Waroux O., Seutin V., Jentsch T.J., Aznar S., Mikkelsen J.D. (2008). Kv7 channels: interaction with dopaminergic and serotonergic neurotransmission in the CNS. J Physiol.

[bib18] Heisler L.K., Cowley M.A., Tecott L.H., Ran W., Low M.J., Smart J.L., Rubinstein M., Tatro J.B., Marcus J.N., Holstege H., Lee C.E., Cone R.D., Elmquist J.K. (2002). Activation of central melanocortin pathways by fenfluramine. Science.

[bib19] Heisler L.K., Jobst E.E., Sutton G.M., Zhou L., Borok E., Thornton-Jones Z., Liu H.Y., Zigman J.M., Balthasar N., Kishi T., Lee C.E., Aschkenasi C.J., Zhang C.Y., Yu J., Boss O., Mountjoy K.G., Clifton P.G., Lowell B.B., Friedman J.M., Horvath T., Butler A.A., Elmquist J.K., Cowley M.A. (2006). Serotonin reciprocally regulates melanocortin neurons to modulate food intake. Neuron.

[bib20] Hoyer D., Hannon J.P., Martin G.R. (2002). Molecular, pharmacological and functional diversity of 5-HT receptors. Pharmacol Biochem Behav.

[bib21] Hoyer D., Clarke D.E., Fozard J.R., Hartig P.R., Martin G.R., Mylecharane E.J., Saxena P.R., Humphrey P.P. (1994). International Union of Pharmacology classification of receptors for 5-hydroxytryptamine (serotonin). Pharmacol Rev.

[bib22] Ioannides-Demos L.L., Proietto J., McNeil J.J. (2005). Pharmacotherapy for obesity. Drugs.

[bib23] Katayama J., Yakushiji T., Akaike N. (1997). Characterization of the K^+^ current mediated by 5-HT1A receptor in the acutely dissociated rat dorsal raphe neurons. Brain Res.

[bib24] Kennett G.A., Ainsworth K., Trail B., Blackburn T.P. (1997). BW 732C86, a 5-HT2B receptor agonist, causes hyperphagia and reduced grooming in rats. Neuropharmacology.

[bib25] Kennett G.A., Wood M.D., Bright F., Trail B., Riley G., Holland V., Avenell K.Y., Stean T., Upton N., Bromidge S., Forbes I.T., Brown A.M., Middlemiss D.N., Blackburn T.P. (1997). SB 242084, a selective and brain penetrant 5-HT2C receptor antagonist. Neuropharmacology.

[bib26] Kilpatrick G.J., Butler A., Burridge J., Oxford A.W. (1990). 1-(M-chlorophenyl)-biguanide, a potent high affinity 5-HT3 receptor agonist. Eur J Pharmacol.

[bib27] Könner A.C., Janoschek R., Plum L., Jordan S.D., Rother E., Ma X., Xu C., Enriori P., Hampel B., Barsh G.S., Khan C.R., Cowley M.A., Ashcroft F.M., Brüning J.C. (2007). Insulin action in AgRP-expressing neurons is required for suppression of hepatic glucose production. Cell Metab.

[bib28] Lovenberg T.W., Erlander M.G., Baron B.M., Racke M., Slone A.L., Siegel B.W., Craft C.M., Burns J.E., Danielson P.E., Sutcliffe J.G. (1993). Molecular cloning and functional expression of 5-HT1E-like rat and human 5-hydroxytryptamine receptor genes. Proc Natl Acad Sci U S A.

[bib29] Lucas J.J., Yamamoto A., Scearce-Levie K., Saudou F., Hen R. (1998). Absence of fenfluramine-induced anorexia and reduced c-fos induction in the hypothalamus and central amygdaloid complex of serotonin 1B receptor knock-out mice. J Neurosci.

[bib30] Makarenko I.G., Meguid M.M., Ugrumov M.V. (2002). Distribution of serotonin 5-hydroxytriptamine 1B (5HT(1B)) receptors in the normal rat hypothalamus. Neurosci Lett.

[bib31] Muraki Y., Yamanaka A., Tsujino N., Kilduff T.S., Goto K., Sakurai T. (2004). Serotonergic regulation of the orexin/hypocretin neurons through the 5-HT_1A_ receptor. J Neurosci.

[bib32] Niswender K.D., Baskin D.G., Schwartz M.W. (2004). Insulin and its evolving partnership with leptin in the hypothalamic control of energy homeostasis. Trends Endocrinol Metab.

[bib33] Peters J.A., Malone H.M., Lambert J.J. (1992). Recent advances in the electrophysiological characterization of 5-HT3 receptors. Trends Pharmacol Sci.

[bib34] Plum L., Ma X., Hampel B., Balthasar N., Coppari R., Münzberg H., Shanabrough M., Burdakov D., Rother E., Janoschek R., Alber J., Belgardt B.F., Koch L., Seibler J., Schwenk F., Fekete C., Suzuki A., Mak T.W., Krone W., Horvath T.L., Ashcroft F.M., Brüning J.C. (2006). Enhanced PIP_3_ signaling in POMC neurons causes K_ATP_ channel activation and leads to diet-sensitive obesity. J Clin Invest.

[bib35] Ramboz S., Saudou F., Amara D.A., Belzung C., Segu L., Misslin R., Buhot M.C., Hen R. (1996). 5-HT1B receptor knock out: behavioural consequences. Behav Brain Res.

[bib36] Ramos E.J.B., Meguid M.M., Campos A.C.L., Coelho J.C.U. (2005). Neuropeptide Y, α-melanocyte-stimulating hormone, and monoamines in food intake regulation. Nutrition.

[bib37] Raymond J.R., Mukhin Y.V., Gelasco A., Turner J., Collinsworth G., Gettys T.W., Grewal J.S., Garnovskaya M.N. (2001). Multiplicity of mechanisms of serotonin receptor signal transduction. Pharmacol Ther.

[bib38] Roseberry A.G., Liu H., Jackson A.C., Cai X., Friedman J.M. (2004). Neuropeptide Y-mediated inhibition of proopiomelanocortin neurons in the arcuate nucleus shows enhanced desensitization in *ob/ob* mice. Neuron.

[bib39] Schiavi G.B., Brunet S., Rizzi C.A., Ladinsky H. (1994). Identification of serotonin 5-HT4 recognition sites in the porcine caudate nucleus by radioligand binding. Neuropharmacology.

[bib40] Schwartz D.H., Hernandez L., Hoebel B.G. (1990). Serotonin release in lateral and medial hypothalamus during feeding and its anticipation. Brain Res Bull.

[bib41] Selkirk J.V., Scott C., Ho M., Burton M.J., Watson J., Gaster L.M., Collin L., Jones B.J., Middlemiss D.N., Price G.W. (1998). SB-224289: a novel selective (human) 5-HT1B receptor antagonist with negative intrinsic activity. Br J Pharmacol.

[bib42] Smith M.A., Hisadome K., Al-Qassab H., Heffron H., Withers D.J., Ashford M.L.J. (2007). Melanocortins and agouti-related protein modulate the excitability of two arcuate nucleus neuron populations by alteration excitability by alteration of resting potassium conductances. J Physiol.

[bib43] Tecott L.H., Sun L.M., Akana S.F., Strack A.M., Lowenstein D.H., Dallman M.F., Julius D. (1995). Eating disorder and epilepsy in mice lacking 5-HT2c serotonin receptors. Nature.

[bib44] Tiligada E., Wilson J.F. (1989). Regulation of alpha-melanocyte-stimulating hormone release from superfused slices of rat hypothalamus by serotonin and the interaction of serotonin with the dopaminergic system inhibiting peptide release. Brain Res.

